# Supplementary material for: Divalent Metal Ions Boost Effect of Nucleic Acids Delivered by Cell-Penetrating Peptides
Source: Cells. 2022 Feb 21;11(4):756. doi: 10.3390/cells11040756 (PMC8870069; doi:10.3390/cells11040756)
Supplement: Supplementary file 1 [file cells-11-00756-s001.zip › cells-1564438-supplementary.pdf]

STable 1. CPPs used, their sequences, charges and number of amino acid residues. All properties of the peptides are modelled and charges or logD values are calculated using MarvinSketch 15.9.14 (ChemAxon Ltd, Budapest, Hungary).

CPP	Alternative name	Sequence	N-terminal fatty acid	Charge (pH=7.4)	Number of amino acid residues
PF3	PepFect 3	AGYLLGKINLKALAALAKKIL	C18	+4	21
PF14	PepFect 14	AGYLLGKLLOOLAAAALOOLL	C18	+5	21
C22-PF14	-	AGYLLGKLLOOLAAAALOOLL	C22	+5	21
PF6	PepFect 6	AGYLLGK[a]INLKALAALAKKIL	C18	+3 (8,9)	21+3K+4 chl.
NF55	NickFect 55	AGYLLGO[b]INLKALAALAKAIL	C18	+3 (3,1)	21
NF70	H52	HHHHYHHGO[b]ILLKALKALAKAIL	C20	+4 (4,6)	22
NF71	H31	HHYHHGO[b]ILLKALKALAKAIL	C18	+4 (5,2)	20
NF72	H82	HHHHHHYHHGO[b]ILLKALKALAKAIL	C20	+4 (5,9)	24

a – to the side chain of indicated lysin residue, one more lysin is added and two lysins are added to it; two chloroquine (chl.) molecules are added to each of 2 lysins

b – synthesis continues from the sidechain, i.e. from δ-amino group, not from α-amino group

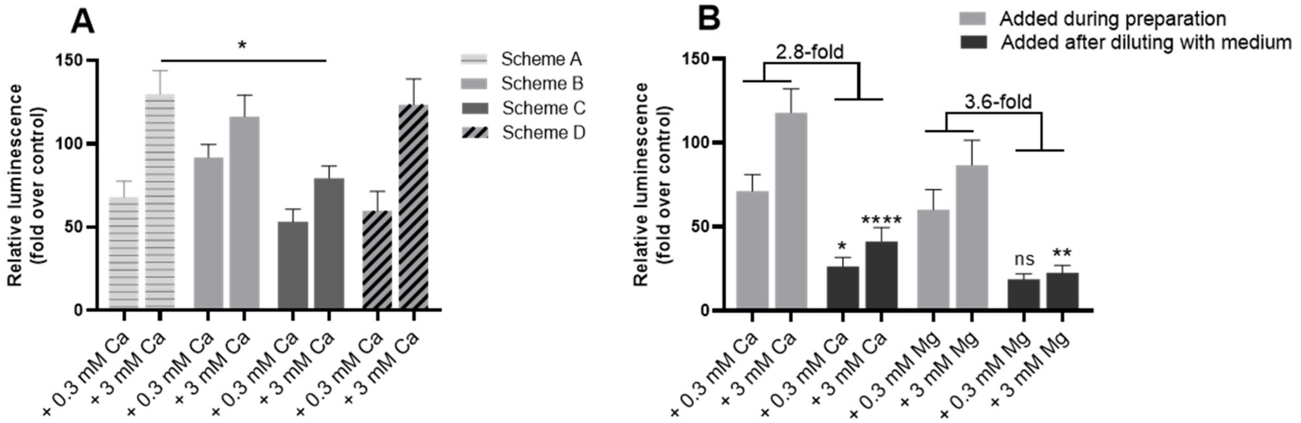

SFig. 1. Effect of SCO-PF14 nanoparticles on luciferase expression in case of different preparation schemes. HeLa pLuc 705 cells were incubated with the solutions containing differently prepared nanoparticles of 100 nM SCO, 500 nM PF14 and 0.3 or 3 mM Ca^{2+}. Scheme A: mixing SCO with PF14, adding Ca^{2+} after 15 min. Scheme B: mixing SCO with PF14 and Ca^{2+}. Scheme C: mixing PF14 with Ca^{2+}, adding SCO after 15 min. Scheme D: mixing SCO with Ca^{2+}, adding PF14 after 15 min. For all schemes, overall incubation time before diluting the nanoparticle-containing solutions with cell culture medium was 30 min. Luminescence was measured after 24 h with microplate reader Infinite M200 PRO (Tecan, Switzerland). Each dataset represents mean + SEM of 4 independent experiments. Data was analyzed by one-way ANOVA with Tukey's test, *p-value < 0.05, **p-value < 0.005, ****p-value < 0.0001, ns – not significant.

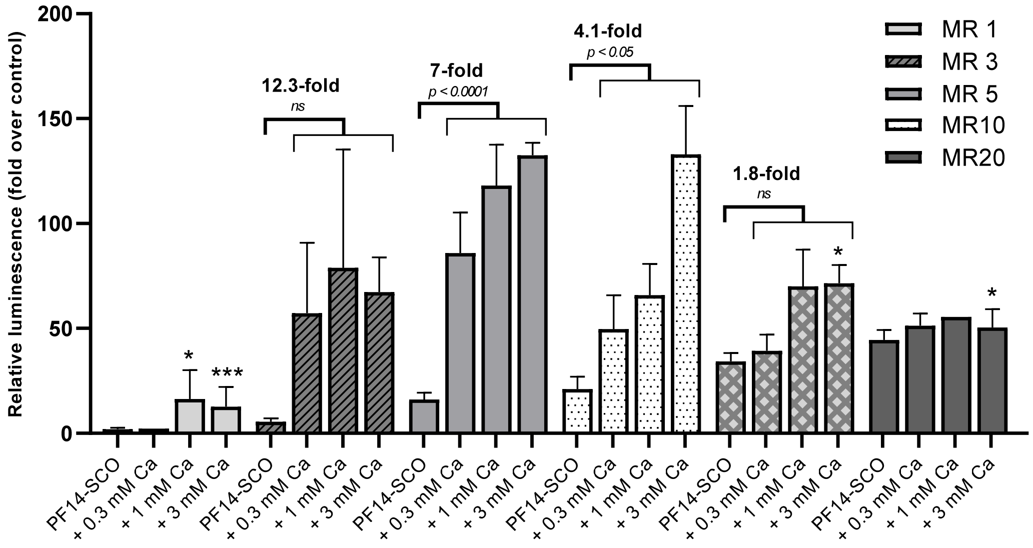

SFig. 2. Effect of SCO-PF14 nanoparticles on luciferase expression in case of different molar ratios. HeLa pLuc 705 cells were incubated with solutions containing nanoparticles of 100 nM SCO, 0.3 or 3 mM Ca^{2+} and 100 nM (MR 1), 300 nM (MR 3), 500 nM (MR 5), 1 µM (MR 10) or 2 µM (MR 20) PF14. Luminescence was measured after 24 h with a microplate reader Infinite M200 PRO (Tecan, Switzerland). Each dataset represents mean + SEM of at least 3 independent experiments performed in triplicates. Data was analyzed by one-way ANOVA with Tukey's test. Asterisks indicate statistically significant difference compared to corresponding dataset in MR 5 group, *p-value < 0.05, ***p-value < 0.0005, ns – not significant.

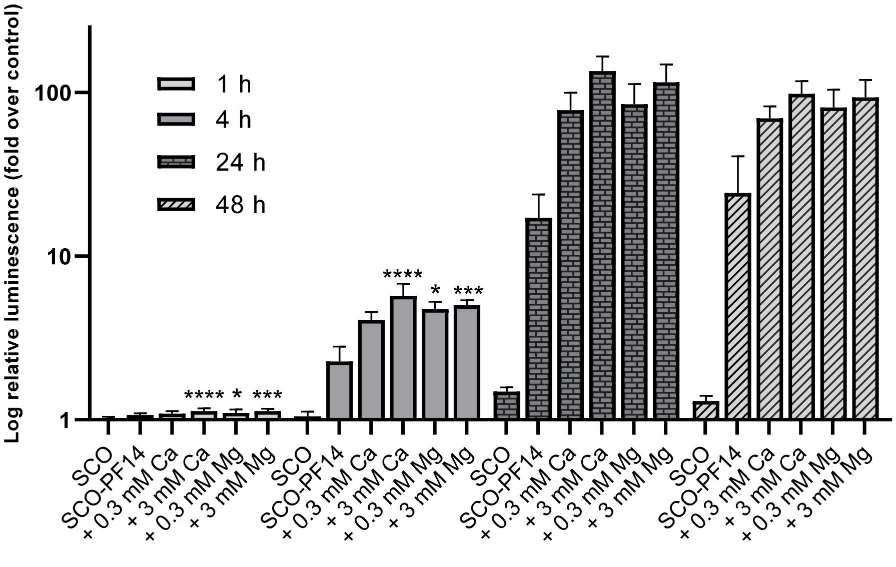

SFig. 3. Time-dependence of SCO-PF14 nanoparticle effect on luciferase expression. HeLa pLuc 705 cells were incubated with nanoparticles of 100 nM SCO-705, 500 nM PF14 and 0.3 or 3 mM Ca^{2+} or Mg^{2+} for 1, 4, 24 or 48 h. Luciferase activity was measured with a microplate reader Infinite M200 PRO (Tecan, Switzerland). Each dataset represents the mean + SEM of 3 independent experiments. Data was analyzed by one-way ANOVA with Tukey's test. Asterisks indicate statistically significant difference compared to corresponding nanoparticle in 24 h incubation group, *p-value < 0.05, ***p-value < 0.0005, ****p-value < 0.0001.

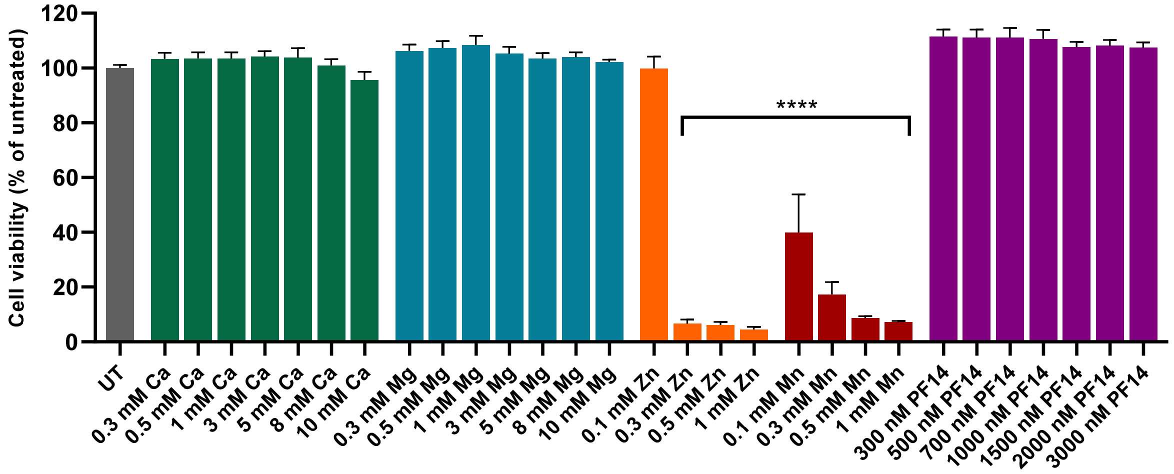

SFig. 4. The effect of PF14 and divalent metal ions on viability of cells. HeLa pLuc 705 cells were incubated for 24 h with solutions containing PF14, Ca^{2+}, Mg^{2+}, Zn^{2+} or Mn^{2+} at different concentrations. Viability of the cells was evaluated using WST-1 assay, and absorption of untreated cells (UT) was taken for 100%. Each dataset represents mean + SEM of at least 3 independent experiments. Data was analyzed by one-way ANOVA with Dunnett's test, asterisks indicate statistically significant difference compared to untreated cells, ****p-value < 0.0001.

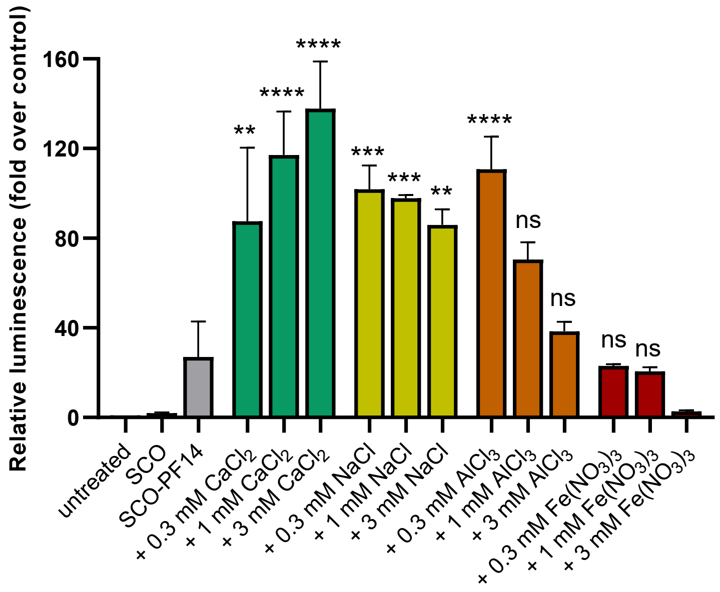

SFig. 5. Effect of calcium, sodium, aluminum and ferric ions on the splicing correction in HeLa pLuc 705 cells. HeLa pLuc 705 cells were incubated with nanoparticles of 100 nM SCO-705, 500 nM PF14 and varying concentrations of respective salts for 24 h. Luciferase activity was measured with a microplate reader Infinite M200 PRO (Tecan, Switzerland). Each dataset represents the mean + SEM of two experiments.
